# Evaluating the *SOS *suicide prevention program: a replication and extension

**DOI:** 10.1186/1471-2458-7-161

**Published:** 2007-07-18

**Authors:** Robert H Aseltine, Amy James, Elizabeth A Schilling, Jaime Glanovsky

**Affiliations:** 1 Division of Behavioral Sciences and Community Health, University of Connecticut Health Center and Institute for Public Health Research, University of Connecticut, 99 Ash Street, MC 7160, East Hartford, Connecticut, 06108, USA; 2 Department of Statistics and Institute for Public Health Research, University of Connecticut, 99 Ash Street, MC 7160, East Hartford, Connecticut, 06108, USA

## Abstract

**Background:**

Suicide is a leading cause of death for children and youth in the United States. Although school based programs have been the principal vehicle for youth suicide prevention efforts for over two decades, few have been systematically evaluated. This study examined the effectiveness of the *Signs of Suicide *(*SOS*) prevention program in reducing suicidal behavior.

**Methods:**

4133 students in 9 high schools in Columbus, Georgia, western Massachusetts, and Hartford, Connecticut were randomly assigned to intervention and control groups during the 2001–02 and 2002–03 school years. Self-administered questionnaires were completed by students in both groups approximately 3 months after program implementation.

**Results:**

Significantly lower rates of suicide attempts and greater knowledge and more adaptive attitudes about depression and suicide were observed among students in the intervention group. Students' race/ethnicity, grade, and gender did not alter the impact of the intervention on any of the outcomes assessed in this analysis.

**Conclusion:**

This study has confirmed preliminary analysis of Year 1 data with a larger and more racially and socio-economically diverse sample. *SOS *continues to be the only universal school-based suicide prevention program to demonstrate significant effects of self-reported suicide attempts in a study utilizing a randomized experimental design. Moreover, the beneficial effects of *SOS *were observed among high school-aged youth from diverse racial/ethnic backgrounds, highlighting the program's utility as a universal prevention program.

**Trial registration:**

clinicaltrials.gov NCT000387855.

## Background

Suicide among young people is one of the most serious public health problems facing the United States. According to the National Center for Health Statistics, in 2003 the suicide rate was 7.3 per 100,000 among youth aged 15–19, making it the third leading cause of death among adolescents [1,2]. Rates of completed suicides, however, mask the actual extent of suicidal behavior among young people. Major epidemiologic studies conducted over the past decade in the US and Canada indicate that thoughts about suicide over the past year have been reported by 17–24% of youth, with actual suicide attempts in the past year reported by 5–10% [3,4]. In the year 2000, 32,655 patients aged 10–19 years were admitted to US hospitals as a result of suicide attempts [5].

In response to this problem, a number of diverse approaches to suicide prevention have been introduced into high school curricula in the past 20 years. Few, however, have been subjected to rigorous evaluation, and those that have been scientifically evaluated have had limited impact, with the benefits of such programs largely confined to temporary improvements in knowledge and more adaptive attitudes about depression and suicidal behavior [6-9]. To date the only universal prevention program to curtail suicidal behavior in a randomized study is *Signs of Suicide *(*SOS*), a program developed by the creators of National Depression Screening Day. Based on evidence from the first year of a 2 year study involving over 2100 students in 5 schools [10], the *SOS *program was added to SAMHSA's National Registry of Effective Programs (NREP) and has been widely adopted, with 675 schools across the country implementing *SOS *during the 2004–05 school year. Findings regarding the benefits of *SOS *were bolstered by a recent randomized trial that found no evidence of iatrogenic effects of a similar suicide screening program [11], thus allaying longstanding concerns about the potential negative effects of suicide prevention programs on emotionally vulnerable youth [12].

This article augments our previous evaluation of the *SOS *program by including data from the second year of the study and expanding the analysis to examine whether the efficacy of the program varies among different types of students. The study was conducted during the 2001–2002 and 2002–2003 school years and involved 4133 students in 213 classrooms in 9 high schools in Hartford, Connecticut, western Massachusetts, and Columbus, Georgia. The recruitment of schools from suburban communities in western Massachusetts in Year 2 added a substantial number of White, middle-class youth to sample. The primary goal of this outcome evaluation was to assess the short-term impact of the program on suicidal behavior, knowledge of and attitudes toward depression and suicide, and help-seeking in a diverse student population.

## Methods

### The intervention

*SOS *is a school-based prevention program developed by Screening for Mental Health, Inc., a non-profit organization in Wellesley, Massachusetts. Fifteen national organizations specializing in youth mental health and suicide prevention serve as sponsors of the *SOS *program, including the American School Counselor Association, National Association of School Psychologists, National Association of Secondary School Principals, and the National Association of Social Workers. *SOS *incorporates two prominent suicide prevention strategies into a single program, combining a curriculum that aims to raise awareness of suicide and its related issues with a brief screening for depression and other risk factors associated with suicidal behavior. The program focuses in particular on two of the most prominent risk factors for suicidal behavior: underlying mental illness, particularly depression, and problematic use of alcohol. In the didactic component of the program, *SOS *promotes the concept that suicide is directly related to mental illness, typically depression, and that it is not a normal reaction to stress or emotional upset [13]. The basic goal of the program is to teach high school students to respond to signs of suicide in themselves and others as an emergency, much as one would react to signs of a heart attack. Youths are taught to recognize the signs and symptoms of suicide and depression and to follow the specific action steps needed to respond to those signs. The objective is to make the action step – ACT – as instinctual a response as the Heimlich maneuver and as familiar an acronym as "CPR." ACT stands for Acknowledge, Care, and Tell. First, ACKNOWLEDGE the signs of suicide that others display and take them seriously. Next, let that person know you CARE about him or her and that you want to help. Then, TELL a responsible adult.

The *SOS *program has been described in detail in previous publications [10,14]. Briefly, the program contains two major components. The first component is a set of teaching materials that include a video, *Friends for Life*, and a discussion guide. The video includes dramatizations depicting the signs of suicidality and depression, recommended ways to react to someone who is depressed and suicidal, as well as interviews with real people whose lives have been touched by suicide. The second component is a screening instrument that is used to assess the potential risk of depression and suicidality. In the first year of this study the Columbia Depression Scale (CDS), a 22-item scale derived from the Diagnostic Interview Schedule for Children IV [15], served as the screening tool. In second year the CDS was replaced with the Brief Screen for Adolescent Depression (BSAD) [16], a shortened version of this screening tool that was also derived from the DISC IV. The screening forms were completed anonymously and scored by the students themselves; a score of 16 or higher on the CDS or 4 or higher on the BSAD was considered a strong indicator of clinical depression and students with such scores were encouraged to seek help immediately from a teacher or counselor, or to approach a trusted adult outside of school. The anonymity of the screening process precluded further evaluation or follow-up of those scoring above the 'at risk' threshold, but the privacy it afforded was considered critical to ensuring an honest assessment of symptoms. Because the screening tools were used exclusively for self-evaluation and were not used to provide baseline data on depression or suicidality, the change in screening forms from year 1 to year 2 would not be expected to have an impact on our analysis.

### Study design

The experimental design consisted of randomized treatment and control groups and posttest-only data collection. In 8 of the 9 participating schools, students were randomly assigned to health classes (Hartford and western Massachusetts) and social studies classes (Columbus) by a computerized scheduling program. (In year 2, only ninth-grade classes were eligible to participate in the Columbus and Hartford sites, because all other grades had received the program during the previous year.) Because the semester in which students were assigned to these half-year classes was determined randomly, all students who took these classes during the first half of the school year were assigned to the treatment group and participated in the program over a 2-day period from October through November. Students who took these classes during the second half of the school year were assigned to the control group and did not participate in the program until after the evaluation was completed. The single exception was a technical-vocational high school in Hartford, where students were clustered in health classes according to their major area of study and where class composition did not change at midyear. For this school, random assignment of classes to both the intervention and the control conditions was achieved by flipping a coin.

Students in both the treatment and the control groups were asked to complete a short questionnaire in a group setting during class time approximately 3 months after implementation of the program. Trained interviewers from the University of Connecticut's Center for Survey Research and Analysis and Columbus State University read aloud the questions to each class, and students recorded their confidential written responses on anonymous questionnaires. Because all data were collected anonymously, a waiver of informed consent was obtained from the University of Connecticut Health Center's Institutional Review Board, which approved all procedures used in this study. Parents were notified in writing about the objectives of the study and were invited to contact their respective schools or the principal investigator (RHA) to ask questions or to withdraw their child from the study. Questionnaires were completed by 4133 of the 4491 students eligible for the study (n = 2094 for the control group, n = 2039 for the treatment group), which resulted in an overall response rate of 92%. Virtually all non-response was due to absences from school as opposed to parental refusal.

As indicated by the demographic profile of the sample (Table 1), these schools provided a racially mixed and economically diverse sample of youths. The addition of schools in western Massachusetts in Year 2 resulted in a substantial increase in the number of White, suburban youth in the sample. Preliminary analyses were conducted to assess the comparability of the intervention group and control group in terms of gender, race/ethnicity, and grade. Chi-square tests revealed no differences in the composition of intervention and control groups for any of these characteristics.

**Table 1:** Demographic characteristics of the sample by location

	**Percent (%)**		
	**Hartford, CT**	**Columbus, GA**	**Massachusetts**

**Race/Ethnicity**			
White, non-Hispanic	5.8	38.5	82.1
Black, non-Hispanic	27.2	36.5	0.8
Hispanic	50.6	8.1	6.0
Multiethnic	8.5	12.4	5.5
Other	8.6	4.6	7.8
	100% (n = 2635)	100% (n = 665)	100% (n = 761)
**Gender**			
Male	48.7	51.6	52.5
Female	51.3	48.4	57.5
	100% (n = 2632)	100% (n = 659)	100% (n = 761)
**Grade**			
Freshman	45.5	100.0	65.6
Sophomore	22.6	0.0	26.3
Junior	15.5	0.0	5.7
Senior	16.3	0.0	2.4
	100% (n = 2593)	100% (n = 665)	100% (n = 752)
**Wave**			
1 – 2002	53.0	100.0	0.0
2 – 2003	47.0	0.0	100.0
	100% (n = 2707)	100% (n = 665)	100% (n = 761)

### Measures and instruments

The questionnaire included items relevant to 3 specific categories of outcome: (1) self-reported suicidal ideation and suicide attempts, (2) knowledge and attitudes about depression and suicide, and (3) help-seeking behavior. The primary endpoint for our study was a single-item measure of self-reported suicide attempts taken from the Centers for Disease Control and Prevention's (CDC) Youth Risk Behavior Survey (YRBS): "During the past 3 months, did you actually attempt suicide (yes or no) [4]?" Suicidal ideation also was assessed with a question taken from the YRBS: "During the past 3 months, did you ever seriously consider attempting suicide (yes or no)?"

The measures of knowledge and attitudes about depression and suicide were adapted from instruments previously used to evaluate school-based suicide prevention programs [6,7,10]. Knowledge of depression and suicide was measured with 10 true/false items that reflect the central themes of the SOS program (e.g., "People who talk about suicide don't really kill themselves"; "Depression is an illness that doctors can treat"). Scores on this variable reflected the number of correct answers. The measure of attitudes toward depression and suicide was an 8-item summary scale that assessed attitudes toward suicidal people and suicidal behaviors (e.g., "If someone really wants to kill him/herself, there is not much I can do about it"; "If a friend told me he/she is thinking about committing suicide, I would keep it to myself"). Responses to these questions ranged from "strongly disagree" to "strongly agree" on a 5-point scale, with higher values indicating more adaptive attitudes about depression and suicide (Cronbach α = .74).

Three questions were used to assess help-seeking behavior. Students were asked whether in the past 3 months, " . . . you received treatment from a psychiatrist, psychologist, or social worker because you were feeling depressed or suicidal (yes or no)"; whether ". . . you talked to some other adult (like a parent, teacher or guidance counselor) because you were feeling depressed or suicidal (yes or no)"; and whether ". . . you talked to an adult about a friend you thought was feeling depressed or suicidal (yes or no)."

Participants who had missing values on any variable in a particular analysis were excluded from that analysis. Although 130 youths assigned to the treatment group did not participate in either of the central elements of the program–the video and depression screening–mainly because of absences from school, they were retained in the analysis so that we could estimate intention-to-treat effects. After exclusions for missing data, the effective sample size for these analyses ranged from 3,837 to 3,899. Descriptive statistics for all dependent variables used in this analysis are shown separately by intervention status in Table 2.

**Table 2:** Descriptive characteristics of measures of suicidal behavior, knowledge, and attitudes

	Control (n = 2094)	Treatment (n = 2039)	Total Sample (N = 4133)	Valid N
Treated for depression/suicidal ideation, %	9.6	9.3	9.4	4024
Talked with adult about depression/suicidal ideation, %	19.5	18.4	18.9	4026
Talked with adult about friends' emotional problems, %	12.4	12.8	12.6	4044
Suicidal ideation during past three months, %	11.5	10.1	10.8	4036
Suicide attempt during past three months, %	4.5	3.0	3.8	4042
Knowledge of depression/suicide, mean (SD)	4.36(1.44)	5.00(1.41)	4.68(1.46)	4029
Attitudes toward depression/suicide, mean (SD)	3.83(0.64)	3.99(0.64)	3.91(0.65)	4044

## Results

To account for the clustered sampling design in which students were nested within classrooms, we used SUDAAN 9.0.1 software [17] to perform regression analyses of intervention effects. SUDAAN was developed to address the complicated variance estimation required in the analysis of data obtained using complex sampling designs, including cluster-correlated data. In our analysis, the effect of exposure to the intervention on the four outcomes in the following three months (Y) was estimated with the following model:

Y = B_0 _+ B_1–9_Controls_1–9 _+ B_10_G_1_

where *G*_1 _is a dummy variable for intervention status; and *Controls*_1–9 _refers to those variables that were significantly related to suicide attempts in preliminary analysis. Controls included dummy variables for race/ethnicity (non-Hispanic Black, Hispanic, multiethnic, and Other race vs. non-Hispanic White), gender (female vs. male), grade (10, 11, and 12 vs. 9), and study wave (2003 vs. 2002).

The effects of the *SOS *program on students' knowledge of and attitudes toward depression and suicide, help-seeking behavior, suicidal ideation, and self-reported suicide attempts are shown in Table 3. For the analysis of attitudes and knowledge, this table shows coefficients from a standard regression model; for suicidal ideation, suicide attempts, and help-seeking behavior, coefficients are derived from logistic regression models. The second row in Table 3 shows the effects of exposure to the *SOS *program on the various outcome measures included in our study. First and most important, the coefficients shown in column 1 of Table 3 indicate that exposure to the *SOS *program was associated with significantly fewer self-reported suicide attempts. The coefficient for the effect of the *SOS *program on attempts is -.47, which when converted to an odds ratio (OR) indicates that the youths in the treatment group were approximately 40% less likely to report a suicide attempt in the past 3 months compared with youths in the control group (OR = *e*^-.47 ^= 0.63). This equates to a 3 month rate of suicide attempts in the *SOS *group of 3.0%, compared to 4.6% among controls.

**Table 3:** Effects of SOS program on students' knowledge of and attitudes toward depression and suicide, help seeking, and suicidal ideation and suicide attempts

	**Attempts**		**Ideation**		**Knowledge**		**Attitudes**		**Help Seeking ** **Treatment**		**Help Seeking ** **Adult**		**Help Seeking ** **Adult/Friend**	
	B	SE	B	SE	B	SE	B	SE	B	SE	B	SE	B	SE

Intercept	-3.17*	0.21	-2.17*	0.14	4.52*	0.06	3.80*	0.03	-2.29*	0.15	-1.72*	0.11	-2.14*	0.14
**SOS Program**	-0.47*	**0.16**	**-0.17**	**0.10**	0.59*	**0.05**	0.16*	**0.03**	**-0.01**	**0.12**	**-0.04**	**0.08**	**0.01**	**0.10**
Female	1.05*	0.22	0.80*	0.12	0.24*	0.04	0.12*	0.02	0.33*	0.12	0.61*	0.11	0.52*	0.11
Hispanic	0.01	0.18	-0.10	0.12	-0.63*	0.06	0.02	0.03	-0.18	0.14	-0.09	0.10	0.01	0.12
Black	-1.44*	0.31	-0.95*	0.16	-0.54*	0.06	-0.01	0.03	-0.36	0.18	-0.27*	0.11	-0.27	0.14
Multiracial	0.13	0.31	0.17	0.17	-0.38*	0.08	-0.06	0.04	0.05	0.21	0.16	0.14	-0.12*	0.19
Other Race	-0.91*	0.46	-0.72*	0.26	-0.49*	0.09	-0.05	0.05	-0.21	0.23	-0.25	0.17	-0.54	0.24
Sophomore	-0.21	0.22	0.01	0.13	0.15*	0.06	-0.08*	0.03	-0.10	0.16	-0.33*	0.12	-0.10	0.13
Junior	-0.37	0.32	-0.41*	0.20	0.13	0.07	0.03	0.04	-0.18	0.22	0.00	0.14	0.01	0.16
Senior	-0.31	0.34	-0.15	0.18	0.22*	0.07	0.04	0.04	0.04	0.18	-0.15	0.14	-0.01	0.16
Wave	-0.42*	0.16	-0.04	0.11	0.33*	0.05	-0.01	0.03	0.12	0.12	0.30*	0.08	0.11	0.09

*p < .05

Note: P-values for intervention effects range from .0000 for knowledge and attitudes about suicide to .0075 for attempts.

Similarly, exposure to the *SOS *program resulted in greater knowledge of depression and suicide and more adaptive attitudes toward these problems (Table 3, columns 3 and 4). The effects of the *SOS *program on knowledge and attitudes were modest in magnitude and resulted in effect sizes of one quarter to one third of a standard deviation (e.g., attitudes: ES = .16/.65 = .25). The effects of the *SOS *program on both attitudes and knowledge remained statistically significant when Holm adjustments were applied to correct for multiple tests that involved these secondary endpoints [18,19]. In contrast, the effects of the *SOS *program on suicidal ideation and help-seeking behavior did not achieve statistical significance.

Finally, modest differences in the outcomes by students' demographic characteristics were observed. For example, the positive gender coefficients in Table 3 indicate that girls have higher rates of suicidal ideation and suicide attempts than boys, greater knowledge of and more adaptive attitudes toward suicide and depression, and higher rates of help-seeking. Significant race differences were also observed, mainly involving contrasts between Blacks and students in the Other category and Whites, with these effects consisting of lower rates of ideation and attempts but also less knowledge of suicide and depression among students in the first 2 categories. Notable age or grade trends were confined to the knowledge measure and favored older students, and base rates of suicide attempts, help-seeking from adults, and knowledge of depression differed by study wave (e.g., the WAVE coefficient). Because of changes in the schools participating in the study in year 2, this effect should be interpreted as an indicator of sample composition rather than a reflection of actual changes in students' attitudes or behavior over this short period of time.

### Differences in intervention effects by students' demographic characteristics

To examine whether the impact of the intervention varied among different types of students, a series of conditional models containing product terms capturing the interaction between exposure to *SOS *and students' race/ethnicity, sex, and grade were estimated for all seven outcome measures in Table 3. Interactions were initially estimated in separate models and subsequently aggregated into a single model for each outcome containing all possible intervention * demographic terms. None of the interaction terms achieved statistical significance at the .05 level. However, it is possible that the failure to detect significant conditional effects is due to inadequate statistical power rather than the absence of substantively meaningful differences in response to the intervention. Although the effects of the program on suicidal ideation and help-seeking were relatively weak, effect sizes for the impact of *SOS *on attitudes, knowledge, and suicide attempts were modest, ranging between .25 and .40 in magnitude. Based on these effect sizes, the power to detect significant interaction effects was calculated to exceed .90 in a sample of 4133 with 213 classrooms using software that accounts for the interdependence of observations in cluster randomized designs [20]. Consequently we conclude that the failure to observe differences in program effects among different types of students is not attributable to insufficient statistical power for these analyses.

## Discussion

This analysis of data from a two year study of 9 schools implementing the *SOS *suicide prevention program confirms and expands Year 1 results that demonstrated the program's efficacy in an urban, economically disadvantaged sample of youth [10]. The present analysis, which is based on a more socially, economically, and geographically diverse group of high school students, found *SOS *to be associated with significantly greater knowledge, more adaptive attitudes about depression and suicide, and most importantly, significantly fewer suicide attempts among intervention youths relative to untreated controls 3 months post-intervention. The magnitude of intervention effects in this more diverse group of students was virtually identical to those reported in the Year 1 results. Moreover, the addition of Year 2 data to the analysis, resulting in a sample of over 4100 youths, provided greater statistical power to examine whether the impact of the *SOS *program is consistent across youths varying in race and ethnicity, gender, and age. These results clearly indicate that the program has broad-based efficacy among high school students of different ages, races and ethnic backgrounds, and for both boys and girls. Furthermore, these results are consistent with findings from previous studies demonstrating the effectiveness of curriculum-based programs in improving knowledge and fostering more favorable attitudes about depression and suicide [6-9].

In contrast to the impact of the program on knowledge, attitudes, and suicidal behavior, *SOS *was not associated with increased help-seeking among emotionally troubled youth. This finding was also observed in our analysis of Year 1 data and was attributed to the composition of the sample given the numerous barriers to help-seeking in urban schools containing large numbers of disadvantaged youth (e.g., staff shortages; poor visibility of mental health resources; students' wariness of confiding in mandated reporters given past contact with social services). We hypothesized that these barriers would be lessened among youth from more suburban, middle-class districts and expected their inclusion in the Year 2 sample to produce positive results, but a separate analysis of the impact of *SOS *on help-seeking among students in the western Massachusetts districts did not reveal any program effects on help seeking. This issue merits further investigation given previously published data from school counselors indicating as much as a three-fold increase in the number of students seeking counseling for depression and suicidality following exposure to SOS [14]. It is possible that the conflicting data on help-seeking reflects a study design issue, as the assignment of classrooms, not schools, to experimental conditions raises the possibility that the control group became contaminated by the intervention. The focus of the *SOS *program makes this a very real possibility: Because youths are encouraged to "ACT" on behalf of a troubled friend, participants in the control groups may have sought help for emotional problems due to their exposure to friends who had participated in the program. If this is the case, a study design that assigns schools to experimental conditions is more likely to produce student reports of help-seeking following exposure to *SOS *that are more consistent with those reported by school counselors.

The contributions of this study must be considered in light of other limitations in addition to the design issues raised above. The effects of this program were observed over a very short post-intervention period. A longer term follow-up of youths exposed to the *SOS *program is necessary to determine whether the observed effects are enduring. Second, pretest measures of the outcomes assessed in this study would add confidence that the assignment of classes to experimental conditions resulted in equivalent groups, and would also allow for comparisons over time within the experimental group to quantify the magnitude of changes in attitudes and behavior that could be attributed to SOS. It should be noted, however, that previous evaluations of suicide prevention programs in which a Solomon four group design was employed to assess the impact of pretest assessments on outcomes at posttest found no impact of the pretest on measures of knowledge and attitudes [9]. Finally, readers may question whether our results are tainted by response bias, particularly the desire of those exposed to the program to provide what they perceive to be the "right answers" or the answers desired by the investigators when responding to survey questions about their attitudes and behavior. However, suicide prevention programs have historically demonstrated very little in the way of efficacy, suggesting that previous research has not been plagued by this type of response bias. There was nothing unique about this sample that would have lead students to have done so here.

## Conclusion

This replication and extension of our 2004 analysis [10] provides confirmation that the *SOS *program is a potent tool for curtailing suicidal behavior among diverse groups of high school-aged youth in the United States. *SOS *continues to be the only universal school-based suicide prevention program for which a reduction in self-reported suicide attempts has been documented with a randomized experimental design. As such it merits serious consideration from teachers, school counselors, and administrators seeking to bolster their school's health curricula and prevention portfolio.

## Competing interests

The author(s) declare that they have no competing interests.

## Authors' contributions

RA conceived of the study and was responsible for writing the manuscript; AJ supervised data collection and management and contributed to writing and revising the manuscript; ES supervised the analysis and contributed to writing and revising the manuscript; JG conducted the analysis.

## Pre-publication history

The pre-publication history for this paper can be accessed here:


